# Global phylogenomic diversity of *Brucella abortus*: spread of a dominant lineage

**DOI:** 10.3389/fmicb.2023.1287046

**Published:** 2023-11-29

**Authors:** Nicolette R. Janke, Charles H. D. Williamson, Kevin P. Drees, Marcela Suárez-Esquivel, Adrian R. Allen, Jason T. Ladner, Christine R. Quance, Suelee Robbe-Austerman, David O’Callaghan, Adrian M. Whatmore, Jeffrey T. Foster

**Affiliations:** ^1^Pathogen and Microbiome Institute, Northern Arizona University, Flagstaff, AZ, United States; ^2^Escuela de Medicina Veterinaria, Universidad Nacional, Heredia, Costa Rica; ^3^Department of Bacteriology, Agri-Food and Biosciences Institute, Belfast, United Kingdom; ^4^USDA-APHIS, National Veterinary Services Laboratories, Ames, IA, United States; ^5^VBIC, INSERM U1047, Université de Montpellier, Nîmes, France; ^6^CNR Brucella, Laboratoire de Microbiologie, CHU Nîmes, Nîmes, France; ^7^WOAH/FAO Reference Laboratory for Brucellosis, Animal and Plant Health Agency, Addlestone, United Kingdom

**Keywords:** brucellosis, phylogeny, *Brucella abortus*, cattle, infectious disease, evolution

## Abstract

*Brucella abortus* is a globally important zoonotic pathogen largely found in cattle hosts and is typically transmitted to humans through contaminated dairy products or contact with diseased animals. Despite the long, shared history of cattle and humans, little is known about how trade in cattle has spread this pathogen throughout the world. Whole genome sequencing provides unparalleled resolution to investigate the global evolutionary history of a bacterium such as *B. abortus* by providing phylogenetic resolution that has been unobtainable using other methods. We report on large-scale genome sequencing and analysis of *B. abortus* collected globally from cattle and 16 other hosts from 52 countries. We used single nucleotide polymorphisms (SNPs) to identify genetic variation in 1,074 *B. abortus* genomes and using maximum parsimony generated a phylogeny that identified four major clades. Two of these clades, clade A (median date 972 CE; 95% HPD, 781–1142 CE) and clade B (median date 150 BCE; 95% HPD, 515 BCE–164 CE), were exceptionally diverse for this species and are exclusively of African origin where provenance is known. The third clade, clade C (median date 949 CE; 95% HPD, 766–1102 CE), had most isolates coming from a broad swath of the Middle East, Europe, and Asia, also had relatively high diversity. Finally, the fourth major clade, clade D (median date 1467 CE; 95% HPD, 1367–1553 CE) comprises the large majority of genomes in a dominant but relatively monomorphic group that predominantly infects cattle in Europe and the Americas. These data are consistent with an African origin for *B. abortus* and a subsequent spread to the Middle East, Europe, and Asia, probably through the movement of infected cattle. We hypothesize that European arrival to the Americas starting in the 15th century introduced *B. abortus* from Western Europe through the introduction of a few common cattle breeds infected with strains from clade D. These data provide the foundation of a comprehensive global phylogeny of this important zoonotic pathogen that should be an important resource in human and veterinary epidemiology.

## Introduction

Despite having limited motility, bacterial pathogens have an uncanny ability to move on continental scales. Human history can in fact be traced using phylogenetic analyses of our associated bacteria, from our emergence out of Africa to our global diaspora, including *Helicobacter pylori* moving with us in our stomachs (Linz et al., 2007), *Vibrio cholerae* in our intestines (Mutreja et al., 2011), *Mycobacterium tuberculosis* in our lungs (Comas et al., 2013; Liu et al., 2018) and *Burkholderia pseudomallei* in trade (Chewapreecha et al., 2017). Bacteria can also disperse with us in animals such as *Bacillus anthracis* in hides and/or hunted animals (Kenefic et al., 2009), *Yersinia pestis* in commensal rodents (Morelli et al., 2010), and *Brucella melitensis* in domesticated goats (Tan et al., 2015). Thus, phylogeographic reconstructions of pathogens can inform us about past human movements and activities and in turn allow us to better understand patterns of disease transmission, dispersal, and host interactions (Keim and Wagner, 2009; Grad and Lipsitch, 2014; Sintchenko and Holmes, 2015).

*Brucella abortus* is one of the world’s most successful pathogens, causing widespread disease in wildlife, livestock, and humans on a global scale (Pappas et al., 2006; Whatmore, 2009). Brucellosis is endemic to much of the world, but the burden of the disease is particularly borne by people and livestock in developing countries (Moreno, 2014). Substantial production losses in cattle occur due to reproductive complications such as abortion, infertility, and decreased milk output (Carvalho Neta et al., 2010). Humans typically contract the disease through contaminated dairy products although occupational exposure occurs in veterinarians, slaughterhouse personnel, and workers involved in animal husbandry. Health impacts on humans are widespread and pronounced, with new brucellosis infections likely in the millions of cases each year and with large sections of the globe, particularly Africa and Asia, poorly sampled but likely containing many undiagnosed cases (Laine et al., 2022). Although *B. abortus* has a broad host range that includes many ruminants such as elk (*Cervus elaphus*) and bison (*Bison bison*) (Schumaker, 2013), swine (*Sus scrofa*) (WOAH, 2023), and goats (*Capra hircus*) (McDermott and Arimi, 2002), domestic cattle remain the most common host (WOAH, 2023). Indeed, this close host-pathogen relationship of *B. abortus* with cattle and its high prevalence in unmanaged herds suggests that the evolutionary history of *B. abortus* may provide unique insights into the history of cattle movements. Then by comparing these genetic patterns in *B. abortus* to cattle genetics, breeding practices, and livestock movements (Pitt et al., 2019; Verdugo et al., 2019; Xia et al., 2023), we can understand how socioeconomic forces and cultural practices have spread this pathogen.

Initial attempts to characterize *B. abortus* involved microbiological and biochemical testing that grouped isolates into eight biovars (bv. 1–7 and 9). However, subsequent work, including the results presented here, indicates that the biovars do not always correspond with distinct genetic groups (Whatmore et al., 2016). Low amounts of genetic diversity have traditionally hampered genetic characterization of *Brucella* taxa (Whatmore, 2009). Fragment based methods such as variable number tandem repeat (VNTR) analysis and multi-locus sequencing have been fundamental to our understanding of the phylogenetic relationships among species and biovars of the genus (Le Fleche et al., 2006; Whatmore et al., 2007). However, homoplasy, lack of resolution at branch tips, and ambiguity at deeper nodes due to limited phylogenetic characters using these methods (Pearson et al., 2009b), suggest a more in-depth approach is required. Comparative approaches using whole genome sequencing provide this needed resolution and form the basis for better understanding evolutionary, epidemiological, and host relationships in *Brucella* (Foster et al., 2009; Wattam et al., 2009, 2014; Audic et al., 2011; Kamath et al., 2016). Early phylogeographic studies in bacteria used a limited number of loci and various genetic approaches that assessed variation within only portions of the genome. Whole genome analyses have become the new standard, particularly in clonal and low diversity bacteria where many loci are required for sufficient power and resolution (Rokas et al., 2003; Pearson et al., 2009b). Single nucleotide polymorphism (SNP) loci are valuable characters for phylogenetic reconstructions due to their evolutionary stability, exhibited by low mutation rates and minimal homoplasy in clonal bacteria (Keim et al., 2004; Achtman, 2008). SNPs have been successfully utilized in phylogeographic comparisons to draw conclusions about the evolutionary history and global dispersal of a variety of pathogens and their diseases e.g., (e.g., Holt et al., 2008; Harris et al., 2010). These studies exhibit the power of whole genome analysis to characterize global diversity in highly clonal bacteria and potentially link the overlapping histories of their host populations.

In this study, we use comparative genomics to interrogate 1,074 genomes of *B. abortus* to determine its evolutionary history and better understand its global movements in cattle and other hosts. Most genomes came from cattle, although at least 16 other animal species, mostly ruminants, were sampled. The large sample size and wide range of locations involved provides a breadth of scope not previously explored in *B. abortus* and an unparalleled opportunity to catalog the extant diversity and phylogeography of this important zoonotic pathogen. Moreover, these data constitute a phylogenetic framework that will be useful in determining the evolutionary significance of new isolates and their relationship to the current phylogenetic framework, and in disease outbreak investigations in an era of global human and animal movement.

## Materials and methods

### Sampling

**FIGURE 1:**
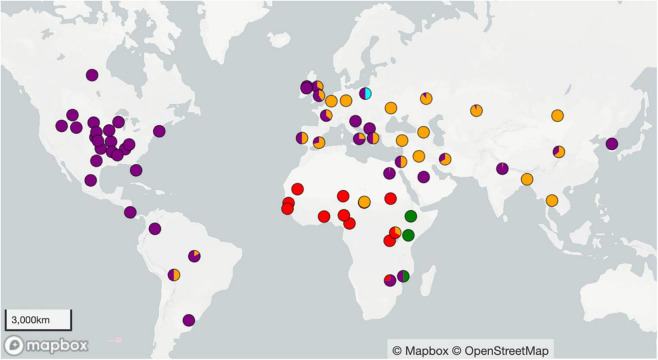
Geographic locations of the 1074 *Brucella abortus* genomes used to construct the phylogenies in Figures 2, 3. Country or state centroid is given except when more specific location information was known. Colors refer to the four clades: clade A is green, B is red, C is orange, D is purple, and light blue is undetermined.

The 1,074 isolates analyzed in this study came from 52 countries across 6 continents (Figure 1 and Supplementary Table 1). Whole genome sequencing data for *B. abortus* isolates were downloaded on June 23, 2023. Paired-end Illumina sequencing data were downloaded from the Sequence Read Archive (Leinonen et al., 2010). Genome assemblies were downloaded from GenBank (Benson et al., 2012) and the Bacterial and Viral Bioinformatics Resource Center (BVBRC).^1^ Genomes were removed from the data set if they aligned poorly to the reference genome (quality breadth <80% in program NASP), were identified as poor quality by BVBRC, or were outliers (i.e., other *Brucella* species). We removed obvious duplicate genomes from the dataset, but retained potential duplicates when we could not determine which genome should take priority. These duplicate genomes served as a doublecheck of our analysis methods, which was confirmed, as all duplicates were identical or highly similar to each other.

The United States (US) was the origin of the largest number of genomes within this study (*n* = 365), with a majority from the Greater Yellowstone Ecosystem (*n* = 264). The United Kingdom (UK) had a relatively large number of isolates (*n* = 93), although as a central repository for strains that have been collected globally, some of these isolates likely originated elsewhere. Ireland, India, Russia, Kazakhstan, Brazil, Egypt, Italy, and Costa Rica were the source of 26–94 isolates each, while the remaining countries were the origin of 1–15 isolates each. The origins of genomes in each lineage and sub-lineage of our phylogeny and basic metadata such as GenBank accession, strain ID, country and collection location, year of collection, host, and phylogenetic lineage are detailed in Supplementary Table 1. Isolates were primarily collected from cattle, but the 16 other hosts included bison (*Bison bison*), elk (*Cervus canadensis*), humans (*Homo sapiens*), water buffalo (*Bubalus bubalis*), goats (*Capra hircus*), swine (*Sus domesticus*), yak (*Bos grunniens*), dog (*Canis lupus familiaris*), camel (*Camelus dromedarius*), llama (*Lama glama*), reindeer (*Rangifer tarandus*), sheep (*Ovis aries*), chamois (*Rupicapra rupicapra*), red fox (*Vulpes vulpes*), cat (*Felis catus*), and mouse (*Mus musculus*). Genomes come from sampling across a wide range of time, spanning 1925 to 2022. Of the genomes in this study, 106 were sequenced in the Brucella II initiative at the Broad Institute (broadinstitute.org), with isolate DNA supplied by the Animal Health and Veterinary Laboratories Agency (AHVLA) [now Animal and Plant Health Agency (APHA)] of the UK, the US Centers for Disease Control and Prevention, or Northern Arizona University. A total of 67 isolates were sequenced by Translational Genomics Research Institute North or the Environmental Genetics and Genomics Laboratory from isolates supplied by US Geological Survey and its collaborators, and 281 samples were sequenced by the collaborations of US Department of Agriculture–National Veterinary Services Laboratory (USDA-NSVL). The remaining genomes were sequenced by various other institutes.

### SNP discovery

Core-genome single nucleotide polymorphisms (SNPs) were called within NASP (Sahl et al., 2016) using paired-end Illumina reads as input. For genome assemblies, paired-end Illumina sequencing data were simulated from publicly-available genome assemblies with ART (MountRainier) (Huang et al., 2012). Reads were aligned to *B. abortus* strain 2308 (GCA_000054005.1) as the reference genome using BWA-MEM (Li, 2013) and SNPs were called with the Unified Genotyper method in GATK (McKenna et al., 2010; DePristo et al., 2011). Positions were removed from the analysis if the depth of coverage was less than ten or if the allele proportion was less than 0.9 for a genome. Duplicated regions of the reference genome were identified with self-alignments using NUCmer (Delcher et al., 2002; Kurtz et al., 2004) and removed from the analysis. A maximum parsimony phylogeny was generated from high-quality, core-genome SNPs (bestsnp.fasta) with the R package phangorn (Schliep, 2011). A consistency index (excluding parsimony uninformative SNPs) and retention index were calculated with phangorn. The consistency index allows one to determine the amount of homoplasy that is occurring within the genetic markers being used in the phylogeny, with values close to 1 indicating limited amounts of homoplasy. To determine the root of the *B. abortus* tree, we first generated a phylogeny that included *B. melitensis* strain 16M (GCA_000740415.1) as an outgroup due to this taxon being sister to *B. abortus* (Wattam et al., 2014). The core genome in these analyses was estimated with the quality breadth metric in NASP (Sahl et al., 2016).

#### Bayesian time-structured coalescent analysis

We plotted the divergence of each tip from the root against the date of sampling (a root-to-tip plot) in the program TempEST. Thus, this temporal signal allowed us to construct a time-structured phylogeny. For the molecular clock estimation, BEAST v1.10.1 (Suchard et al., 2018) was used to generate a time-structured phylogeny including only a subset (*n* = 607) of the GenBank genomes with known isolation year. For this analysis, NASP was run again, as described above, to generate a core SNP matrix specific for this subset of isolates. The matrix included only variable positions, but the BEAST XML input file was modified to specify the number of invariant sites, by nucleotide, in the *B. abortus* genomes. Six different combinations of molecular clock and coalescent models were evaluated using path-sampling and stepping-stone marginal likelihood estimation approaches (Lartillot and Philippe, 2006; Fan et al., 2011; Xie et al., 2011). Each model combination was run in duplicate, with one billion Markov chain Monte Carlo steps, and sampling parameters and trees assessed every 100,000 generations to ensure independent convergence of the chains. The log files were combined with LogCombiner v1.10.1 and assessed with Tracer v1.7.1. The first 50,000,000 iterations were discarded as burn-in. As the relaxed clock models did not converge, even when using a subset of the genomes, model selection was based only on strict clock model results, in which all the tree models’ effective sample sizes (ESS) were ≥236. The best fit model combination was a strict molecular clock, along with the Bayesian Coalescent Skyline tree prior with 10 categories (Drummond, 2005).

## Results

**FIGURE 2:**
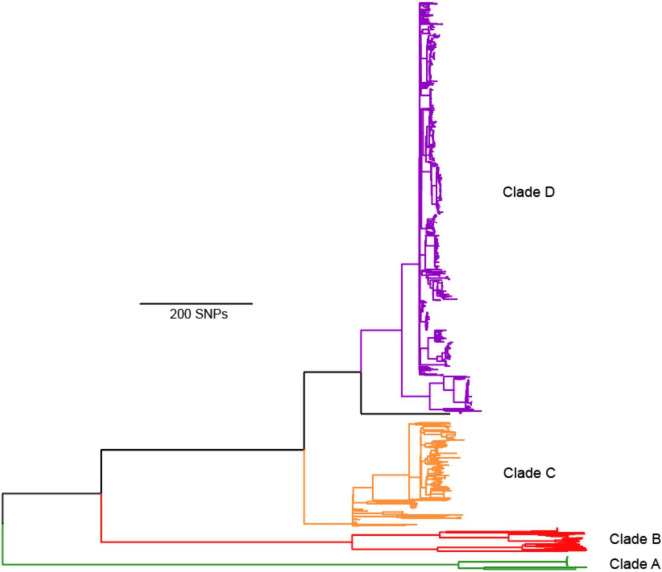
Maximum parsimony phylogeny of 1074 *Brucella abortus* genomes rooted with *B. melitensis* 16M outgroup. We define four major lineages A, B, C, D, which correspond to the four main MLST groups of Whatmore et al. (2016), but as A, B, C2, and C1, respectively. Full details of isolate origins and other metadata are found in Supplementary Table 1.

The first phylogenetic tree illustrates the genomic comparisons of all *B. abortus* genomes and is rooted with the outgroup (*B. melitensis* 16M) (Figure 2). A total of 2,053 SNPs separated *B. melitensis* 16M from the *B. abortus* isolates. The core genome in this analysis was 1,619,280 nucleotide positions, with SNPs at 13,723 positions. Homoplasy was low, with a consistency index (excluding parsimony uninformative SNPs) of 0.96, and a retention index of 0.99. We then analyzed only the *B. abortus* genomes, which involved 1,629,697 nucleotide positions in the core genome, SNPs at 11,797 positions, a consistency index (excluding parsimony uninformative SNPs) of 0.97, and a retention index of 0.99.

**FIGURE 3:**
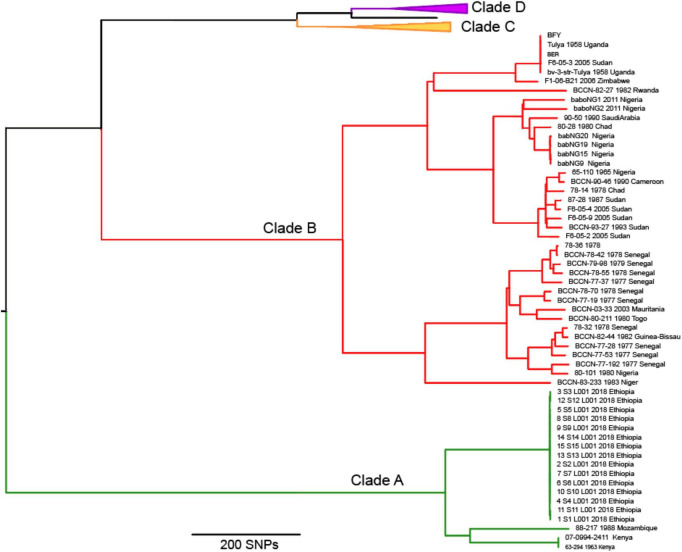
Maximum parsimony phylogeny of 56 *Brucella abortus* genomes from clades A and B, all but one have African origins, including the strain Tulya from Uganda, the reference strain for biovar 3. Clades C and D have been collapsed to allow for details of clades A and B to be visualized.

The phylogeny revealed what we consider as four major clades of *B. abortus*, with two clades (A and B) composed almost entirely of isolates of African origin. For isolates with known origin, 52 of 53 came from African countries (Figure 3), the sole exception being an isolate from Saudi Arabia, which has an established connection to Africa from infected livestock with brucellosis via historical trade (Foster et al., 2018). The most notable member of these two African clades is the biovar 3 reference strain Tulya, which was isolated from a Ugandan cow in 1958. The distribution of biovars within the African clades is also striking, with biovars 3, 6, and the previously classified biovar 7 predominating [biovar 7 is no longer in usage (Garin-Bastuji et al., 2014)]; these biovars account for nearly all of the African isolates where biovar is known. Despite containing only a small fraction of the total genomes in our analysis, more genetic variation exists in the 56 genomes from the African clades than exists in all of the other *B. abortus* genomes, as indicated by the relatively long branch lengths to and within these clades (Figures 2, 3).

**FIGURE 4:**
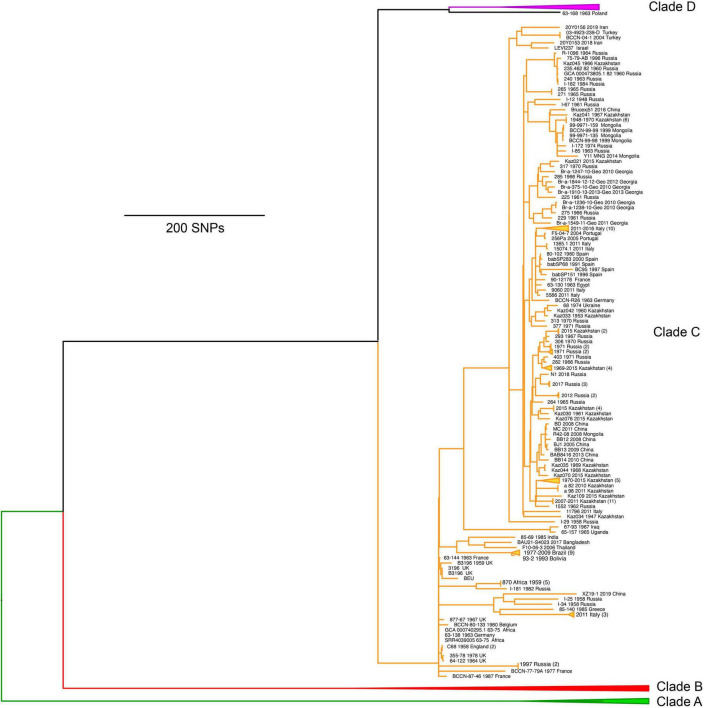
Maximum parsimony phylogeny of *Brucella abortus* genomes from clade C, which largely have origins in Asia. Clades A, B, and D have been collapsed to allow for details of clade C to be visualized.

The third major lineage, identified as clade C (corresponding to C2 of MLSA) (Whatmore et al., 2016; Shevtsov et al., 2023), contained isolates of diverse origins but came almost entirely from Europe and Asia (Figure 4). Relative to the diversity within the African clades, the *B. abortus* genomes from clade C exhibited minimal genetic diversity but were substantially more diverse than the relatively monomorphic clade D (see below). Clade C can be divided into two broad subclades, with one subclade containing notable isolates such as the reference strains for biovar 5 (strain B3196), biovar 6 (strain 870), and biovar 9 (strain C68). In contrast, the other subclade contains no reference strains but does include a large number of isolates predominantly from Asia, particularly countries with extensive sampling such as Kazakhstan, Russia, and China, and to a lesser extent countries such as Brazil, Italy, Mongolia, and Georgia. The diversity and geographic distributions of clade C have been previously described and molecular dating indicates the arrival of this subclade into Kazakhstan in the 19th or early 20th century (Shevtsov et al., 2023). One unusual finding from Italy is that despite strains sharing a common host, water buffalo, Italian herds are infected with strains from both clades C and D, suggesting two separate introductions and two distinct lineages have remained despite control efforts (Garofolo et al., 2013, 2017). Clade C seems likely to have been imported with infected water buffalo from Asia and clade D was likely acquired locally from infected cattle as the two species interact on some farms. Of note, when extensive sampling has been conducted for a study focused on a single location over a short time frame, those genomes will generally cluster together and are depicted as triangles for the collapsed branches (Figure 4).

**FIGURE 5:**
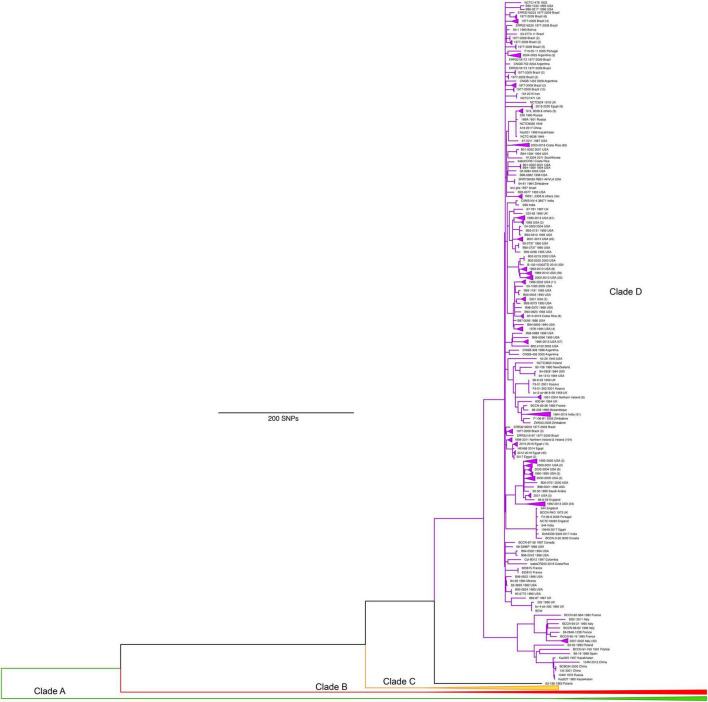
Maximum parsimony phylogeny of *Brucella abortus* genomes from clade D, which largely have origins in W. Europe, and North and South America. Clades A, B, and C have been collapsed to allow for details of clade D to be visualized.

Clade D contains the largest number of genomes (*n* = 837) from six continents but surprisingly little genetic diversity and is largely composed of biovars 1, 2, and 4 (Figures 2, 5). Clade D contains multiple subclades, several with striking geographic localization of closely related genomes, as was also seen in clade C. These clusters were associated with sampling of infected livestock (primarily cattle herds and water buffalo) and included five clusters associated with the Yellowstone region (Kamath et al., 2016), and additional clusters from Costa Rica (Suarez-Esquivel et al., 2020), Northern Ireland (Allen et al., 2020), Brazil (Pereira et al., 2023), Texas (this study), and Italy (Garofolo et al., 2017). This geographic clustering of closely related strains demonstrates the low diversity of most brucellosis outbreaks. Interestingly, some of these clusters contain isolates that were collected over several decades, indicating that limited differentiation and circulation occurs over these time scales. Interspersed among these clusters were samples from other US states (26 states sampled) and diverse international locations such as the South American countries of Argentina, Bolivia, Brazil, and Trinidad and Tobago, European countries including France, Kosovo, Poland, Portugal, Spain, and the UK, as well as Mexico, New Zealand, and Zimbabwe. The overall global pattern for clade D is the spread of this lineage over the past several centuries followed by limited and local differentiation. However, the connectedness of global trade allows for some isolates to spread between distant locations. Finer scale details for all genomes in this study can be found online: https://microreact.org/project/t3oxnHhmZrAhtuw6mamutc-b-abortusglobal-phylogenomic-diversity.

**FIGURE 6:**
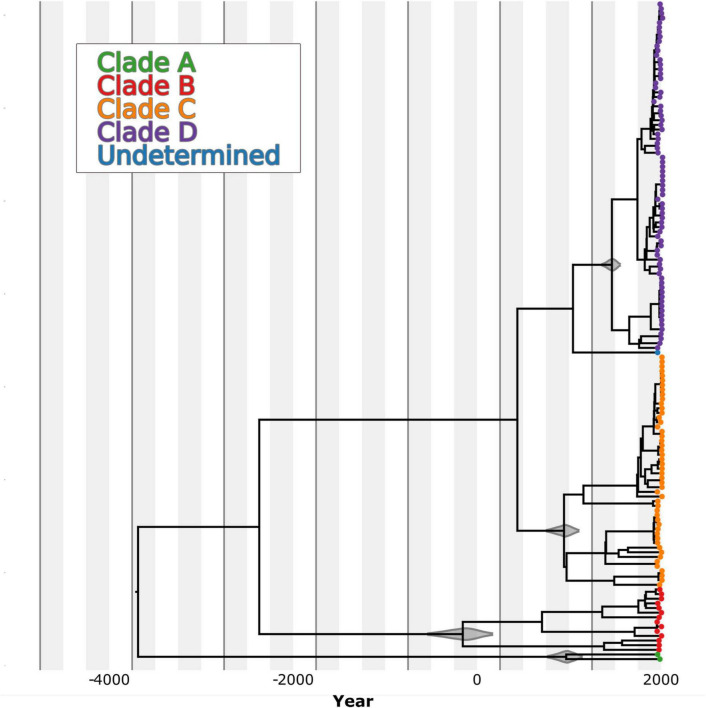
Time-structured maximum clade credibility (MCC) phylogenetic tree of 607 *B. abortus* genomes. The branches are color-coded according to the MLST clade clustering pattern, which also corresponds to the grouping based on the geographic origin of the genomes. The violin plots over the nodes show the 95% highest posterior distribution of the time to the most common recent ancestor (MRCA) from each one of the clusters.

Our results indicate that the most recent common ancestor (MRCA) of *B. abortus* as a species occurred approximately 3726 BCE (median estimate date 3683 BCE, 95% HPD 4657–2871 BCE; Table 1), with an estimated mean substitution (subs) rate of 6.49 × 10^–8^ subs/sites/year. This rate is highly similar to a rate estimate for *B. melitensis* of 9.3 × 10^–8^ subs/sites/year (Long et al., 2023) as well as our rate estimates from *B. abortus* spread in Costa Rica of 8.28 × 10^–8^ subs/sites/year (95% HPD interval: 2.8 × 10^–8^–1.7 × 10^–7^ (Suarez-Esquivel et al., 2020), which was estimated with an uncorrelated relaxed clock model and a skyline tree. This shows consistency in the estimation of the substitution rate for *Brucella* in general despite the model used for the analysis. The B clade, which includes genomes from Africa, is predicted as the oldest *B. abortus* clade based on the available sequences, followed by clades C, A and D (Figure 6). Nonetheless, both clades A and B are poorly sampled and unsampled diversity likely remains that would make these divergence times earlier.

**TABLE 1:** Time of most recent common ancestor of *Brucella abortus* and clades from BEAST analysis.

	Mean	Median	95% HPD* interval
TMCRA	3727 BCE	3683 BCE	4657 BCE, 2871 BCE
Clade A	965 CE	972 CE	781 CE, 1142 CE
Clade B	164 BCE	150 BCE	516 BCE, 164 BCE
Clade C	941 CE	949 CE	766 CE, 1102 CE
Clade D	1464 CE	1468 CE	1367 CE, 1553 CE

*HPD, high posterior density, a Bayesian version of a confidence interval. Tree topology and clade branching depicted in Figure 6.

## Discussion

Homoplasy was minimal within our trees, increasing confidence in the topologies. Homoplasy can be the consequence of directional selection or horizontal gene transfer, and the low levels seen here in *B. abortus* are indicative a largely clonal population of organisms undergoing minimal recombination or convergent evolution (as also occurs in *Mycobacterium tuberculosis*; (Comas et al., 2013) and where nearly all genetic variation is created by mutations. This is consistent with *B. abortus* being a clonal intracellular pathogen that has had limited genetic exchange with other bacteria in its recent evolution.

### African origin of *B. abortus*

We posit that Africa is the likely origin of *B. abortus.* Clades C and D are nested within clades A and B containing the African strains. We note that this differs from the “basal clade” interpretation often mistaken as indicative of a species’ origin or for clades containing ancestral traits, which is an especially common misconception in trees with poorly sampled taxa in a sister group (Crisp and Cook, 2005). Also supporting an African origin is that strains from clades A and B have the highest amounts of genetic diversity within the species, and high levels of genetic diversity often indicate a species’ geographic origin (Pearson et al., 2009a). Similar phylogeographic findings have supported an African origin for another important bacterial taxon of zoonotic pathogens—the *Mycobacterium tuberculosis* complex (Comas et al., 2013). If *B. abortus* has had a limited number of hosts and similar selective pressures, one would not expect substantial differences in substitution rates among lineages of *B. abortus*. Moreover, all of the genomes in our analyses came from contemporary samples within the past few decades. We are thus left trying to explain the relatively long branches for clades A and B; it is likely that substantial diversity remains to be discovered within these two clades and that missing diversity is most likely in Africa. These findings suggest the emergence of *B. abortus* in Africa and from there an expansion into Europe and Asia (clade C), and later to the Americas (clade D).

Understanding the relationship between *B. abortus* and cattle and their mutual global spread is a complex issue, complicated by imprecise estimates of the timing of both cattle and *Brucella* evolution. Furthermore, matching our proposed African origin of *B. abortus* with cattle evolution on the continent is made more challenging by the uncertain timing and spread of cattle in Africa. Taurine (*Bos taurus*) cattle domestication occurred in the Fertile Crescent in roughly 8500 BCE, along with the domestication of goats, sheep, and pigs slightly earlier (Zeder, 2008). A second cattle domestication event, the zebu or indicine species (*Bos indicus*) occurred in the Indus Valley in roughly 6500 BCE (Loftus et al., 1994). Although the archeological and molecular evidence are much debated, both cattle species subsequently spread to Africa and interbred with each other as well as with wild aurochs (*Bos primigenius*) (Felius et al., 2014; Pitt et al., 2019; Verdugo et al., 2019). As a result, African cattle are genetically diverse (Troy et al., 2001; Hanotte, 2002; Decker et al., 2014) but also provide numerous opportunities and timepoints for *B. abortus* to emerge. Brucellosis in African cattle, particularly in sub-Saharan Africa, is widespread and remains a substantial burden for animal production (McDermott and Arimi, 2002; Ducrotoy et al., 2017). Sampling and genotyping of strains from these animals will better inform the movement and history of *B. abortus* on this continent and we are likely only seeing a small proportion of its diversity. Khames et al. (2017) described new diversity within *B. abortus* isolates from Algeria, although we are not certain where these samples would fall within our phylogenies as the genomes are not yet available. We are cautious, however, about overinterpreting the phylogenetic data with respect to geography (Crisp et al., 2011). Relating phylogenetic patterns with biogeographic patterns is even more difficult to disentangle in *B. abortus* and other intracellular pathogens because of the essential host-pathogen relationship, the potential effects of domestication and human transport of livestock, complex cattle and human histories, and the ability of pathogens to switch hosts. A broad range of ungulates and other wildlife species are suitable hosts for *B. abortus*, although most sampling to date has found genotypes from clade D (Simpson et al., 2021).

Moreno et al. (2002) proposed that the *Brucella* genus began to diverge 20–25 million years ago concurrent with speciation of ovine, caprine and bovine hosts from their ancestors. However, results from Foster et al. (2009) using rough approximations of mutation rates indicated that while the divergence of species within the *Brucella* genus is more recent, although we must note, estimated this divergence far earlier than our present study. Our molecular dating presented here suggest even more recent emergence of the core/classical *Brucella* (Whatmore and Foster, 2021). It is tempting to speculate that animal domestication in the Middle East allowed for the emergence of several *Brucella* species in livestock. Long et al. (2023) used molecular dating of *B. melitensis* and concluded that this species diverged from *B. abortus* early in the domestication of sheep and goats. Our results from *B. abortus* are consistent with these findings but are not certain as to the exact timing of when and where *B. abortus* diverged from *B. melitensis*. Our time-based reconstruction indicates that clade B diverged from the *B. abortus* common ancestor sometime from 515 BC to 164 AD. Taken with the findings we describe herein, it seems plausible therefore that wild ancestors of domesticated bovines or other ruminants in Africa were afflicted by brucellosis and that host specific evolution of *B. abortus* and its subsequent diversification predated domestication. However, to adequately address this question would require a denser sampling of both wildlife and wild and domesticated bovine species infected with *B. abortus*, and ideally, generating genomes from ancient DNA from bones from animals or humans infected with brucellosis (Kay et al., 2014; Long et al., 2023).

#### Movement of *B. abortus* out of Africa

Our trees do not inform us as to the likely path of movement of *B. abortus* out of Africa but based on current distributions it appears that the lineages went in two directions. Clade C originated roughly in 972 CE (range 766–1102 CE), spread primarily into the Middle East and then into the rest of Asia, with some movement into Europe as well. The spread into Asia may have occurred along the Silk Road, as has been proposed for *B. melitensis* (Pisarenko et al., 2018), although Shevtsov et al. (2023) suggest that movement into Kazakhstan might have been much more recent, in the past 100–200 years. Clade C is well distributed across Eurasia, from present day western Europe to China, but represents considerably lower amounts of genetic diversity compared to the African lineages. This reduced diversity may result from a variety of factors. Sampling bias and a reduced number of available genomes may underestimate the true diversity, although the 60 genomes from this clade are dispersed over a large extent of Eurasia, which should aid in capturing a representative level of the extant diversity. Additionally, our data on the major African lineages is indicative that even small sample numbers from a specific geographic region can provide useful information on pathogen genetic diversity relative to other regions. We speculate that the movement of *B. abortus* across Eurasia may be tied to the domestication of wild aurochs into taurine cattle in the Middle East around 10,000 years ago (Zeder, 2008) and the subsequent spread of agriculture—and infected animals—across these two continents (Achilli et al., 2008; Decker et al., 2014). More recent movements of livestock out of Africa and into the Middle East have been connected to Arab trade in the region (Foster et al., 2018; Holzer et al., 2021), and may also be connected to animal importation into Saudi Arabia during the Hajj each year. It is plausible that non-domesticated animals could have aided the movement of *B. abortus* across Eurasia, but we are unaware of likely hosts to have allowed for this wide dispersal. Modern reports of the spread of zoonotic disease also indicate that human mediated movement of infected animals underpins much of disease spread (Christley et al., 2005; Bigras-Poulin et al., 2006; Rautureau et al., 2010).

In contrast to the diversity and widespread distribution of clade C, clade D is largely restricted to Western Europe and the Americas. Despite the many inconsistencies with biovars and frequent lack of correspondence to genetic groupings (Whatmore, 2009), biovars 3, 6, 7, and 9 comprise clades A, B, and C, and clade D is comprised of biovars 1, 2, and 4. The latter may potentially be attributed to the difficulty of transporting cattle across oceans in the past and that this was done solely by European colonists, a historical fact given additional molecular credence by the finding that greater than 50% of cattle mtDNA variation can be partitioned among the continents (Bradley et al., 1996). Along with the presence of genomes from the US in clade D, these biovar data hint as to how *B. abortus* reached the Americas (detailed below). The spread of clade D as a dominant lineage is similar to the clonal expansions of other notable pathogens such as *Bacillus anthracis*, *Yersinia pestis*, and *Francisella tularensis* (Keim and Wagner, 2009).

#### The New World

The majority of the genomes found in clade D are from the Americas, principally from the US; although sampling bias in our isolate collection undoubtedly plays a role. When biotyped, nearly all of these samples are from biovars 1, 2, and 4, although isolates from these three biovars do not always group together. Despite the 837 genomes and many subclades within this lineage, the branching is shallow and overall SNP variation with these genomes is very limited compared to the rest of *B. abortus*. This suggests a relatively recent introduction of genetically monomorphic *B. abortus* strains into the Americas from Western Europe, which is consistent with our molecular dating estimates of the emergence of clade D of ∼1467 AD. Tellingly, embedded within this mainly American group of genomes and sub-lineages includes closely related genomes from Europe. We hypothesize that the historical events that likely lie at the root of this bacterial movement are the introduction of infected livestock into the Americas by European colonists as early as the 15^th^ and 16^th^ centuries. More European isolates as well as historic isolates would allow us to more conclusively address this question, however it is striking to observe the close genetic relationship between genomes from North America and the UK and South America and Portugal/Spain. Given the past colonial histories of these territories, we believe the latter observations strengthen our hypothesis. Furthermore, this same pattern of associations between European countries and their colonies appears to be occurring in *B. melitensis* in goats and sheep with the introduction of the Americas clade into the Western hemisphere, likely soon after the colonization of the New World (Al Dahouk et al., 2007; Tan et al., 2015; Pisarenko et al., 2018).

#### A wider diaspora beyond the Americas?

We have presented larger patterns in our trees that suggest wide-ranging movement of *B. abortus* globally. We do not go into all of the details of every genome and its origin, but it is worth noting that many have complex histories. The continued global movement of people, animals, and goods, and in fact the greater global connectivity allows for widespread movement of pathogens such as *B. abortus*. Embedded within clade D are genomes from diverse locations such as India, Mozambique, New Zealand, Saudi Arabia, and Zimbabwe. This suggests that the spread of *B. abortus* out of Europe to the Americas was part of a wider diaspora involving global trade from Europe with many other territories or that these strains were introduced from American sources at later dates. A similar pattern of worldwide spread was observed for another important zoonotic veterinary pathogen, *Mycobacterium bovis*, where the central role of Britain in trading cattle globally during the expansion of the British Empire underpinned the spread of this disease (Smith et al., 2011). Moreover, British farmers played a leading role in developing many modern cattle breeds (Decker et al., 2009). The Hereford breed in particular has been an important genetic stock used in the foundation and improvement of many beef cattle breeds across North and South America, Australia and New Zealand (Porter, 1991).

### Within the United States

Within clade D, we observe at least eight sub-clades that are specific to particular regions or outbreaks (Figure 5). Five of these sub-clades are linked to the Yellowstone area, with isolates originating from both domestic and wildlife species (Kamath et al., 2016)—a complex multi-host epidemiology that has been previously noted in traditional veterinary and molecular based studies (Higgins et al., 2012; Cross et al., 2013). Three of these sub-clades also contain samples from outside this region, reflecting the connection of infected cattle throughout the country with Yellowstone as the recipient or source of infected animals. The three other subclades containing mostly US genomes have some geographic localization such as clusters of samples in Arkansas, Kansas, Missouri, Texas, and Mississippi, but the overall pattern is a homogenized population throughout the country apparently spread by the cattle trade.

## Conclusion

Whole genome phylogenetic analysis of global *B. abortus* genomes suggests Africa as the origin of *B. abortus*. Subsequent movement out of Africa likely went in two directions, one into Western Europe and the other into Asia. Strains in the Americas appear to have originated from the introduction of a limited genetic stock of bacteria, likely originating in cattle from Europe during the period of European colonization of the North and South American continents.

The large number of isolates from the US serves to illustrate the potential usefulness of genome sequencing to further understand the dynamics of disease spread within territories. In particular, the observation that the Greater Yellowstone Area has been subject to at least five separate pathogen introductions affecting multiple animal hosts illustrates the power of this method to provide novel and meaningful epidemiological insights. With denser sampling of animals over longer time periods, there is the potential to gain even greater understanding of local epidemics using a phylodynamic approach such as has been illustrated before with a variety of human and animal pathogens (Volz et al., 2009; Biek et al., 2012; Harris et al., 2013).

Having a global phylogeny such as the one we present here is also of great potential use in determining likely infection sources for new outbreaks in an age of globalization and long-distance livestock transport. What we present herein is a foundation on which to build more detailed surveys of other locales to expand the usefulness of this resource. Increasing the collection of isolates and genomes so that future phylogenies are more representative of the global distribution of *B. abortus* is essential to our understanding the movement and evolution of these important bacteria as well as for taking One Health actions for animal and human health.

## Data availability statement

The datasets presented in this study can be found in online repositories. The names of the repository/repositories and accession number(s) can be found in the article/Supplementary material.

## Author contributions

NJ: Formal analysis, Writing – original draft. CW: Data curation, Formal analysis, Methodology, Writing – review and editing. KD: Data curation, Formal analysis, Methodology, Writing – original draft. MS-E: Data curation, Formal analysis, Visualization, Writing – review and editing. AA: Formal analysis, Methodology, Writing – review and editing. JL: Formal analysis, Methodology, Writing – review and editing. CQ: Data curation, Resources, Writing – review and editing. SR-A: Data curation, Resources, Writing – review and editing. DO’C: Conceptualization, Funding acquisition, Investigation, Resources, Writing – review and editing. AW: Conceptualization, Funding acquisition, Investigation, Resources, Writing – review and editing. JF: Conceptualization, Data curation, Formal analysis, Funding acquisition, Investigation, Methodology, Project administration, Resources, Supervision, Visualization, Writing – original draft, Writing – review and editing.

## Footnotes

1
https://www.bv-brc.org/

## Supplementary material

Supplementary Table 1 Summary data for genomes making up major clades A–D, including GenBank Accession Number or SRA identifier for sequencing data. Genome information including strain name, country of origin, year of collection, and host largely come from the National Center for Biotechnology Information (NCBI) GenBank BioSample Attributes metadata fields.

## References

[B1] Achilli A. Olivieri A. Pellecchia M. Uboldi C. Colli L. Al-Zahery N. (2008). Mitochondrial genomes of extinct aurochs survive in domestic cattle. *Curr. Biol.* 18 R157–R158. 10.1016/j.cub.2008.01.019 18302915

[B2] Achtman M. (2008). Evolution, population structure, and phylogeography of genetically monomorphic bacterial pathogens. *Annu. Rev. Microbiol.* 62 53–70. 10.1146/annurev.micro.62.081307.162832 18785837

[B3] Al Dahouk S. Le Fleche P. Nockler K. Jacques I. Grayon M. Scholz H. C. (2007). Evaluation of *Brucella* MLVA typing for human brucellosis. *J. Microbiol. Methods* 69 137–145. 10.1016/j.mimet.2006.12.015 17261338

[B4] Allen A. R. Milne G. Drees K. Presho E. Graham J. Mcadam P. (2020). Genomic epizootiology of a *Brucella abortus* outbreak in Northern Ireland (1997–2012). *Infect. Genet. Evol.* 81:104235. 10.1016/j.meegid.2020.104235 32035245

[B5] Audic S. Lescot M. Claverie J.-M. Cloeckaert A. Zygmunt M. (2011). The genome sequence of Brucella pinnipedialis B2/94 sheds light on the evolutionary history of the genus Brucella. *BMC Evol. Biol.* 11:200. 10.1186/1471-2148-11-200 21745361 PMC3146883

[B6] Benson D. A. Cavanaugh M. Clark K. Karsch-Mizrachi I. Lipman D. J. Ostell J. (2012). GenBank. *Nucleic Acids Res.* 41 D36–D42. 10.1093/nar/gkr1202 23193287 PMC3531190

[B7] Biek R. O’Hare A. Wright D. Mallon T. Mccormick C. Orton R. J. (2012). Whole genome sequencing reveals local transmission patterns of *Mycobacterium bovis* in sympatric cattle and badger populations. *PLoS Pathog.* 8:e1003008. 10.1371/journal.ppat.1003008 23209404 PMC3510252

[B8] Bigras-Poulin M. Thompson R. A. Chriel M. Mortensen S. Greiner M. (2006). Network analysis of Danish cattle industry trade patterns as an evaluation of risk potential for disease spread. *Preventive Vet. Med.* 76 11–39. 10.1016/j.prevetmed.2006.04.004 16780975

[B9] Bradley D. G. Machugh D. E. Cunningham P. Loftus R. T. (1996). Mitochondrial diversity and the origins of African and European cattle. *Proc. Natl. Acad. Sci. U.S.A.* 93 5131–5135. 10.1073/pnas.93.10.5131 8643540 PMC39419

[B10] Carvalho Neta A. V. Mol J. P. S. Xavier M. N. Paixão T. A. Lage A. P. Santos R. L. (2010). Pathogenesis of bovine brucellosis. *Vet. J.* 184 146–155. 10.1016/j.tvjl.2009.04.010 19733101

[B11] Chewapreecha C. Holden M. T. G. Vehkala M. Välimäki N. Yang Z. Harris S. R. (2017). Global and regional dissemination and evolution of *Burkholderia pseudomallei*. *Nat. Microbiol.* 2:16263. 10.1038/nmicrobiol.2016.263 28112723 PMC5300093

[B12] Christley R. M. Robinson S. E. Lysons R. French N. P. (2005). “Network analysis of cattle movement in Great Britain,” in *Proceedings of a meeting held at Nairn, Inverness*, Scotland.

[B13] Comas I. Coscolla M. Luo T. Borrell S. Holt K. E. Kato-Maeda M. (2013). Out-of-Africa migration and Neolithic coexpansion of *Mycobacterium tuberculosis* with modern humans. *Nat. Genet.* 45 1176–1182. 10.1038/ng.2744 23995134 PMC3800747

[B14] Crisp M. D. Cook L. G. (2005). Do early branching lineages signify ancestral traits? *Trends Ecol. Evol.* 20 122–128. 10.1016/j.tree.2004.11.010 16701355

[B15] Crisp M. D. Trewick S. A. Cook L. G. (2011). Hypothesis testing in biogeography. *Trends Ecol. Evol.* 26 66–72. 10.1016/j.tree.2010.11.005 21146898

[B16] Cross P. C. Maichak E. J. Brennan A. Scurlock B. M. Henningsen J. Luikart G. (2013). An ecological perspective on *Brucella abortus* in the western United States. *Rev. Sci. Tech.* 32 79–87. 10.20506/rst.32.1.2184 23837367

[B17] Decker J. E. Mckay S. D. Rolf M. M. Kim J. Molina Alcalá A. Sonstegard T. S. (2014). Worldwide patterns of ancestry, divergence, and admixture in domesticated cattle. *PLoS Genet.* 10:e1004254. 10.1371/journal.pgen.1004254 24675901 PMC3967955

[B18] Decker J. E. Pires J. C. Conant G. C. Mckay S. D. Heaton M. P. Chen K. (2009). Resolving the evolution of extant and extinct ruminants with high-throughput phylogenomics. *Proc. Natl. Acad. Sci. U.S.A.* 106 18644–18649. 10.1073/pnas.0904691106 19846765 PMC2765454

[B19] Delcher A. L. Phillippy A. Carlton J. Salzberg S. L. (2002). Fast algorithms for large-scale genome alignment and comparison. *Nucleic Acids Res.* 30 2478–2483. 10.1093/nar/30.11.2478 12034836 PMC117189

[B20] DePristo M. A. Banks E. Poplin R. Garimella K. V. Maguire J. R. Hartl C. (2011). A framework for variation discovery and genotyping using next-generation DNA sequencing data. *Nat. Genet.* 43 491–498. 10.1038/ng.806 21478889 PMC3083463

[B21] Drummond A. J. (2005). Bayesian coalescent inference of past population dynamics from molecular sequences. *Mol. Biol. Evol.* 22 1185–1192. 10.1093/molbev/msi103 15703244

[B22] Ducrotoy M. Bertu W. J. Matope G. Cadmus S. Conde-Álvarez R. Gusi A. M. (2017). Brucellosis in Sub-Saharan Africa: Current challenges for management, diagnosis and control. *Acta Trop.* 165 179–193. 10.1016/j.actatropica.2015.10.023 26551794

[B23] Fan Y. Wu R. Chen M.-H. Kuo L. Lewis P. O. (2011). Choosing among partition models in Bayesian phylogenetics. *Mol. Biol. Evol.* 28 523–532. 10.1093/molbev/msq224 20801907 PMC3002242

[B24] Felius M. Beerling M.-L. Buchanan D. S. Theunissen B. Koolmees P. A. Lenstra J. A. (2014). On the history of cattle genetic resources. *Diversity* 6 705–750.

[B25] Foster J. T. Beckstrom-Sternberg S. M. Pearson T. Beckstrom-Sternberg J. S. Chain P. S. G. Roberto F. F. (2009). Whole genome-based phylogeny and divergence of the genus *Brucella*. *J. Bacteriol.* 191 2864–2870. 10.1128/JB.01581-08 19201792 PMC2668414

[B26] Foster J. T. Walker F. M. Rannals B. D. Hussain M. H. Drees K. P. Tiller R. V. (2018). African lineage *Brucella melitensis* isolates from Omani livestock. *Front. Microbiol.* 8:2702. 10.3389/fmicb.2017.02702 29379492 PMC5775276

[B27] Garin-Bastuji B. Mick V. Le Carrou G. Allix S. Perrett L. L. Dawson C. E. (2014). Examination of taxonomic uncertainties surrounding *Brucella abortus* bv. 7 by phenotypic and molecular approaches. *Appl. Environ. Microbiol.* 80 1570–1579. 10.1128/AEM.03755-13 24362435 PMC3957594

[B28] Garofolo G. Di Giannatale E. De Massis F. Zilli K. Ancora M. Camma C. (2013). Investigating genetic diversity of *Brucella abortus* and *Brucella melitensis* in Italy with MLVA-16. *Infect. Genet. Evol.* 19 59–70. 10.1016/j.meegid.2013.06.021 23831636

[B29] Garofolo G. Di Giannatale E. Platone I. Zilli K. Sacchini L. Abass A. (2017). Origins and global context of *Brucella abortus* in Italy. *BMC Microbiol.* 17:28. 10.1186/s12866-017-0939-0 28152976 PMC5290641

[B30] Grad Y. H. Lipsitch M. (2014). Epidemiologic data and pathogen genome sequences: a powerful synergy for public health. *Genome Biol.* 15:538. 10.1186/s13059-014-0538-4 25418119 PMC4282151

[B31] Hanotte O. (2002). African pastoralism: genetic imprints of origins and migrations. *Science* 296 336–339. 10.1126/science.1069878 11951043

[B32] Harris S. R. Cartwright E. J. P. Török M. E. Holden M. T. G. Brown N. M. Ogilvy-Stuart A. L. (2013). Whole-genome sequencing for analysis of an outbreak of meticillin-resistant Staphylococcus aureus: a descriptive study. *Lancet Infect. Dis.* 13 130–136. 10.1016/S1473-3099(12)70268-2 23158674 PMC3556525

[B33] Harris S. R. Feil E. J. Holden M. T. G. Quail M. A. Nickerson E. K. Chantratita N. (2010). Evolution of MRSA during hospital transmission and intercontinental spread. *Science* 327 469–474. 20093474 10.1126/science.1182395 PMC2821690

[B34] Higgins J. Stuber T. Quance C. Edwards W. H. Tiller R. V. Linfield T. (2012). Molecular epidemiology of *Brucella abortus* isolates from cattle, elk, and bison in the United States, 1998 to 2011. *Appl. Environ. Microbiol.* 78 3674–3684. 10.1128/AEM.00045-12 22427502 PMC3346378

[B35] Holt K. E. Parkhill J. Mazzoni C. J. Roumagnac P. Weill F. X. Goodhead I. (2008). High-throughput sequencing provides insights into genome variation and evolution in *Salmonella* Typhi. *Nat. Genet.* 40 987–993. 10.1038/ng.195 18660809 PMC2652037

[B36] Holzer K. El-Diasty M. Wareth G. Abdel-Hamid N. H. Hamdy M. E. R. Moustafa S. A. (2021). Tracking the Distribution of Brucella abortus in Egypt Based on Core Genome SNP Analysis and In Silico MLVA-16. *Microorganisms* 9:1942. 10.3390/microorganisms9091942 34576838 PMC8469952

[B37] Huang W. Li L. Myers J. R. Marth G. T. (2012). ART: A next-generation sequencing read simulator. *Bioinformatics* 28 593–594. 22199392 10.1093/bioinformatics/btr708 PMC3278762

[B38] Kamath P. L. Foster J. T. Drees K. P. Luikart G. Quance C. Anderson N. J. (2016). Genomics reveals historic and contemporary transmission dynamics of a bacterial disease among wildlife and livestock. *Nat. Commun.* 7:11448. 10.1038/ncomms11448 27165544 PMC4865865

[B39] Kay G. L. Sergeant M. J. Giuffra V. Bandiera P. Milanese M. Bramanti B. (2014). Recovery of a medieval *Brucella melitensis* genome using shotgun metagenomics. *mBio* 5 e1337–e1314. 10.1128/mBio.01337-14 25028426 PMC4161259

[B40] Keim P. Van Ert M. N. Pearson T. Vogler A. J. Huynh L. Y. Wagner D. M. (2004). Anthrax molecular epidemiology and forensics: using the appropriate marker for different evolutionary scales. *Infect. Genet. Evol.* 4 205–213. 10.1016/j.meegid.2004.02.005 15450200

[B41] Keim P. S. Wagner D. M. (2009). Humans and evolutionary and ecological forces shaped the phylogeography of recently emerged diseases. *Nat. Rev. Microbiol.* 7 813–821. 10.1038/nrmicro2219 19820723 PMC2794044

[B42] Kenefic L. J. Pearson T. Okinaka R. T. Schupp J. M. Wagner D. M. Ravel J. (2009). Pre-columbian origins for North American anthrax. *PLoS One* 4:e4813. 10.1371/journal.pone.0004813 19283072 PMC2653229

[B43] Khames M. Mick V. De Miguel M. J. Girault G. Conde-Álvarez R. Khelef D. (2017). The characterization of *Brucella* strains isolated from cattle in Algeria reveals the existence of a *B. abortus* lineage distinct from European and Sub-Saharan Africa strains. *Vet. Microbiol.* 211 124–128. 10.1016/j.vetmic.2017.10.008 29102107

[B44] Kurtz S. Phillippy A. Delcher A. L. Smoot M. Shumway M. Antonescu C. (2004). Versatile and open software for comparing large genomes. *Genome Biol.* 5 R12. 10.1186/gb-2004-5-2-r12 PMC395750 14759262

[B45] Laine C. G. Scott H. M. Arenas-Gamboa A. M. (2022). Human brucellosis: Widespread information deficiency hinders an understanding of global disease frequency. *PLoS Negl. Trop. Dis.* 16:e0010404. 10.1371/journal.pntd.0010404 35580076 PMC9113565

[B46] Lartillot N. Philippe H. (2006). Computing Bayes factors using thermodynamic integration. *Syst. Biol.* 55 195–207. 16522570 10.1080/10635150500433722

[B47] Le Fleche P. Jacques I. Grayon M. Al Dahouk S. Bouchon P. Denoeud F. (2006). Evaluation and selection of tandem repeat loci for a *Brucella* MLVA typing assay. *BMC Microbiol.* 6:9. 10.1186/1471-2180-6-9 16469109 PMC1513380

[B48] Leinonen R. Sugawara H. Shumway M. International Nucleotide Sequence Database Collaboration. (2010). The Sequence Read Archive. *Nucleic Acids Res.* 39 D19–D21. 21062823 10.1093/nar/gkq1019 PMC3013647

[B49] Li H. (2013). Aligning sequence reads, clone sequences and assembly contigs with BWA-MEM. *arXiv* [Preprint]. 10.48550/arXiv.1303.3997

[B50] Linz B. Balloux F. Moodley Y. Manica A. Liu H. Roumagnac P. (2007). An African origin for the intimate association between humans and *Helicobacter* pylori. *Nature* 445 915–918. 10.1038/nature05562 17287725 PMC1847463

[B51] Liu Q. Ma A. Wei L. Pang Y. Wu B. Luo T. (2018). China’s tuberculosis epidemic stems from historical expansion of four strains of *Mycobacterium tuberculosis*. *Nat. Ecol. Evol.* 2 1982–1992. 10.1038/s41559-018-0680-6 30397300 PMC6295914

[B52] Loftus R. T. Machugh D. E. Bradley D. G. Sharp P. M. Cunningham P. (1994). Evidence for two independent domestications of cattle. *Proc. Natl. Acad. Sci.* 91 2757–2761. 8146187 10.1073/pnas.91.7.2757 PMC43449

[B53] Long G. S. Hider J. Duggan A. T. Klunk J. Eaton K. Karpinski E. (2023). A 14th century CE *Brucella melitensis* genome and the recent expansion of the Western Mediterranean clade. *PLoS Pathog.* 19:e1011538. 10.1371/journal.ppat.1011538 37523413 PMC10414615

[B54] McDermott J. J. Arimi S. M. (2002). Brucellosis in sub-Saharan Africa: epidemiology, control and impact. *Vet. Microbiol.* 90 111–134. 12414138 10.1016/s0378-1135(02)00249-3

[B55] McKenna A. Hanna M. Banks E. Sivachenko A. Cibulskis K. Kernytsky A. (2010). The Genome Analysis Toolkit: a MapReduce framework for analyzing next-generation DNA sequencing data. *Genome Res.* 20 1297–1303. 10.1101/gr.107524.110 20644199 PMC2928508

[B56] Morelli G. Song Y. Mazzoni C. J. Eppinger M. Roumagnac P. Wagner D. M. (2010). *Yersinia pestis* genome sequencing identifies patterns of global phylogenetic diversity. *Nat. Genet.* 42 1140–1143. 10.1038/ng.705 21037571 PMC2999892

[B57] Moreno E. (2014). Retrospective and prospective perspectives on zoonotic brucellosis. *Front. Microbiol.* 5:213–213. 10.3389/fmicb.2014.00213 24860561 PMC4026726

[B58] Moreno E. Cloeckaert A. Moriyon I. (2002). *Brucella* evolution and taxonomy. *Vet. Microbiol.* 90 209–227. 10.1016/s0378-1135(02)00210-9 12414145

[B59] Mutreja A. Kim D. W. Thomson N. R. Connor T. R. Lee J. H. Kariuki S. (2011). Evidence for several waves of global transmission in the seventh cholera pandemic. *Nature* 477 462–465. 10.1038/nature10392 21866102 PMC3736323

[B60] Pappas G. Papadimitriou P. Akritidis N. Christou L. Tsianos E. V. (2006). The new global map of human brucellosis. *Lancet Infect. Dis.* 6 91–99. 16439329 10.1016/S1473-3099(06)70382-6

[B61] Pearson T. Giffard P. Beckstrom-Sternberg S. Auerbach R. Hornstra H. Tuanyok A. (2009a). Phylogeographic reconstruction of a bacterial species with high levels of lateral gene transfer. *BMC Biol.* 7:78. 10.1186/1741-7007-7-78 19922616 PMC2784454

[B62] Pearson T. Okinaka R. T. Foster J. T. Keim P. (2009b). Phylogenetic understanding of clonal populations in an era of whole genome sequencing. *Infect. Genet. Evol.* 9 1010–1019. 10.1016/j.meegid.2009.05.014 19477301

[B63] Pereira C. R. Neia R. C. Silva S. B. Williamson C. H. D. Gillece J. D. O’callaghan D. (2023). Comparison of *Brucella abortus* population structure based on genotyping methods with different levels of resolution. *J. Microbiol. Methods* 211:106772. 10.1016/j.mimet.2023.106772 37343840

[B64] Pisarenko S. V. Kovalev D. A. Volynkina A. S. Ponomarenko D. G. Rusanova D. V. Zharinova N. V. (2018). Global evolution and phylogeography of *Brucella melitensis* strains. *BMC Genomics* 19:353. 10.1186/s12864-018-4762-2 29747573 PMC5946514

[B65] Pitt D. Sevane N. Nicolazzi E. L. Machugh D. E. Park S. D. E. Colli L. (2019). Domestication of cattle: Two or three events? *Evol. Applic.* 12 123–136. 10.1111/eva.12674 PMC6304694 30622640

[B66] Porter V. (1991). *Cattle - A handbook to the breeds of the world.* London: A & C Black Ltd.

[B67] Rautureau S. Dufour B. Durand B. (2010). Vulnerability of animal trade networks to the spread of infectious diseases: A methodological approach applied to evaluation and emergency control strategies in cattle, France, 2005. *Transbound. Emerg. Dis.* 58 110–120. 10.1111/j.1865-1682.2010.01187.x 21159152

[B68] Rokas A. Williams B. L. King N. Carroll S. B. (2003). Genome-scale approaches to resolving incongruence in molecular phylogenies. *Nature* 425 798–804. 14574403 10.1038/nature02053

[B69] Sahl J. W. Lemmer D. Travis J. Schupp J. M. Gillece J. D. Aziz M. (2016). NASP: an accurate, rapid method for the identification of SNPs in WGS datasets that supports flexible input and output formats. *Microb. Genom.* 2 e000074. 10.1099/mgen.0.000074 28348869 PMC5320593

[B70] Schliep K. P. (2011). phangorn: phylogenetic analysis in R. *Bioinformatics* 27 592–593. 10.1093/bioinformatics/btq706 21169378 PMC3035803

[B71] Schumaker B. (2013). Risks of *Brucella abortus* spillover in the Greater Yellowstone area. *Rev. Sci. Tech.* 32 71–77. 10.20506/rst.32.1.2185 23837366

[B72] Shevtsov A. Cloeckaert A. Berdimuratova K. Shevtsova E. Shustov A. V. Amirgazin A. (2023). Brucella abortus in Kazakhstan, population structure and comparison with worldwide genetic diversity. *Front. Microbiol.* 14:1106994. 10.3389/fmicb.2023.1106994 37032899 PMC10073595

[B73] Simpson G. Thompson P. N. Saegerman C. Marcotty T. Letesson J. J. De Bolle X. (2021). Brucellosis in wildlife in Africa: a systematic review and meta-analysis. *Sci. Rep.* 11:5960. 10.1038/s41598-021-85441-w 33727580 PMC7966391

[B74] Sintchenko V. Holmes E. C. (2015). The role of pathogen genomics in assessing disease transmission. *BMJ* 350 h1314. 10.1136/bmj.h1314 25964672

[B75] Smith N. H. Berg S. Dale J. Allen A. Rodriguez S. Romero B. (2011). European 1: A globally important clonal complex of *Mycobacterium bovis*. *Infect. Genet. Evol.* 11 1340–1351. 10.1016/j.meegid.2011.04.027 21571099

[B76] Suarez-Esquivel M. Hernandez-Mora G. Ruiz-Villalobos N. Barquero-Calvo E. Chacon-Diaz C. Ladner J. T. (2020). Persistence of *Brucella abortus* lineages revealed by genomic characterization and phylodynamic analysis. *PLoS Negl. Trop. Dis.* 14:e0008235. 10.1371/journal.pntd.0008235 32287327 PMC7182279

[B77] Suchard M. A. Lemey P. Baele G. Ayres D. L. Drummond A. J. Rambaut A. (2018). Bayesian phylogenetic and phylodynamic data integration using BEAST 1.10. *Virus Evol.* 4 vey016. 10.1093/ve/vey016 29942656 PMC6007674

[B78] Tan K.-K. Tan Y.-C. Chang L.-Y. Lee K. W. Nore S. S. Yee W.-Y. (2015). Full genome SNP-based phylogenetic analysis reveals the origin and global spread of *Brucella melitensis*. *BMC Genomics* 16:93. 10.1186/s12864-015-1294-x 25888205 PMC4409723

[B79] Troy C. S. Machugh D. E. Bailey J. F. Magee D. A. Loftus R. T. Cunningham P. (2001). Genetic evidence for near eastern origins of European cattle. *Nature* 410 1088–1091. 10.1038/35074088 11323670

[B80] Verdugo M. P. Mullin V. E. Scheu A. Mattiangeli V. Daly K. G. Maisano Delser P. (2019). Ancient cattle genomics, origins, and rapid turnover in the Fertile Crescent. *Science* 365 173–176. 10.1126/science.aav1002 31296769

[B81] Volz E. M. Kosakovsky Pond S. L. Ward M. J. Leigh Brown A. J. Frost S. D. W. (2009). Phylodynamics of infectious disease epidemics. *Genetics* 183 1421–1430. 19797047 10.1534/genetics.109.106021 PMC2787429

[B82] Wattam A. R. Foster J. T. Mane S. P. Beckstrom-Sternberg S. M. Beckstrom-Sternberg J. M. Dickerman A. W. (2014). Comparative phylogenomics and evolution of the Brucellae reveal a path to virulence. *J. Bacteriol.* 196 920–930. 10.1128/JB.01091-13 24336939 PMC3957692

[B83] Wattam A. R. Williams K. P. Snyder E. E. Almeida N. F. Jr. Shukla M. Dickerman A. W. (2009). Analysis of ten *Brucella* genomes reveals evidence for horizontal gene transfer despite a preferred intracellular lifestyle. *J. Bacteriol.* 191 3569–3579. 10.1128/JB.01767-08 19346311 PMC2681906

[B84] Whatmore A. M. (2009). Current understanding of the genetic diversity of *Brucella*, an expanding genus of zoonotic pathogens. *Infect. Genet. Evol.* 9 1168–1184. 10.1016/j.meegid.2009.07.001 19628055

[B85] Whatmore A. M. Foster J. T. (2021). Emerging diversity and ongoing expansion of the genus *Brucella*. *Infect. Genet. Evol.* 92:104865. 10.1016/j.meegid.2021.104865 33872784

[B86] Whatmore A. M. Koylass M. S. Muchowski J. Edwards-Smallbone J. Gopaul K. K. Perrett L. L. (2016). Extended multilocus sequence analysis to describe the global population structure of the genus *Brucella*: phylogeography and relationship to biovars. *Front. Microbiol.* 7:2049. 10.3389/fmicb.2016.02049 28066370 PMC5174110

[B87] Whatmore A. M. Perrett L. L. Macmillan A. P. (2007). Characterisation of the genetic diversity of *Brucella* by multilocus sequencing. *BMC Microbiol.* 7:34. 10.1186/1471-2180-7-34 17448232 PMC1877810

[B88] WOAH (2023). *Manual of Diagnostic Tests and Vaccines for Terrestrial Animals*, 12th Edn. Paris: World Organization for Animal Health.

[B89] Xia X. Qu K. Wang Y. Sinding M.-H. S. Wang F. Hanif Q. (2023). Global dispersal and adaptive evolution of domestic cattle: a genomic perspective. *Stress Biol.* 3:8. 10.1007/s44154-023-00085-2 37676580 PMC10441868

[B90] Xie W. Lewis P. O. Fan Y. Kuo L. Chen M.-H. (2011). Improving marginal likelihood estimation for Bayesian phylogenetic model selection. *Syst. Biol.* 60 150–160. 21187451 10.1093/sysbio/syq085 PMC3038348

[B91] Zeder M. A. (2008). Domestication and early agriculture in the Mediterranean Basin: Origins, diffusion, and impact. *Proc. Natl. Acad. Sci. U.S.A.* 105 11597–11604. 10.1073/pnas.0801317105 18697943 PMC2575338

